# Effectiveness of Transesophageal Echocardiography in Preventing Thromboembolic Complications Before Cardioversion: A Narrative Review

**DOI:** 10.7759/cureus.48149

**Published:** 2023-11-02

**Authors:** Prateek Jain, Vishwesh Patel, Yashaswi Patel, Jawairiya Rasool, Siddharth Kamal Gandhi, Priyansh Patel

**Affiliations:** 1 Department of Internal Medicine, Maulana Azad Medical College, Delhi, IND; 2 Department of Internal Medicine, M.P. Shah Government Medical College, Jamnagar, IND; 3 Department of Internal Medicine, Government Medical College, Surat, Surat, IND; 4 Department of Internal Medicine, Dow International Medical College, Karachi, PAK; 5 Department of Internal Medicine, Medical College Baroda, Vadodara, IND

**Keywords:** left atrial appendage thrombus, electrical cardioversion, post cardioversion, atrial fibrillation, transesophageal echocardiography (tee)

## Abstract

Atrial fibrillation (AFib) is one of the most prevalent irregular heartbeats that doctors encounter. Clinicians typically pursue two main approaches for treatment, namely, controlling the heart rate and managing the heart rhythm. Under the rhythm control approach, AFib is addressed through cardioversion, which is achieved either with medications termed pharmacological cardioversion (PCV) or via an electrical shock termed electric cardioversion (ECV). While ECV proves instrumental in AFib management, it carries its own risk factors, potentially leading to blood clot-related complications such as embolic strokes. To counteract this potential downside, a well-established strategy involves the utilization of transesophageal echocardiography (TEE) to identify possible embolic sources before initiating cardioversion. The goal of this systematic review is to highlight the role of TEE in preempting embolic occurrences following ECV during the management of AFib. After conducting a thorough search of databases, namely, PubMed, PubMed Central, and Medline, a total of 36 studies were selected for this review article. Following a comprehensive evaluation of these studies, it was concluded that TEE plays a pivotal role in preventing thromboembolic complications during ECV for AFib. However, it is important to note that further research is needed to delve deeper into this matter. While existing evidence underscores its efficacy, additional investigation is needed to address this subject matter comprehensively.

## Introduction and background

Atrial fibrillation (AFib) is the most common long-lasting irregular heartbeat that doctors come across in their practice. It affects about 0.4% of the general population, which adds up to around 2.2 million Americans. One of the major consequences of AFib is its link to clot-related issues and strokes [1]. Stroke is one of the top causes of mortality worldwide [2,3]. This heart condition raises the risk of stroke by four to six times (going up to 15 times if an individual has a history of rheumatic heart disease), making it a significant factor for strokes in the elderly and the leading cause of embolic stroke [1]. According to the Global Burden of Disease, the estimated prevalence of AFib is up to 33.5 million individuals, as it affects 2.5-3.5% of the population in several countries [4]. For patients experiencing symptoms due to irregular heart rhythms such as AFib, who require a way to control their heart rhythm, a key step in treatment is cardioversion. This can be done either by delivering a controlled electric shock using direct current (called electrical cardioversion, ECV) or by using medications for restoring regular heart rhythm (known as pharmacological cardioversion, PCV). This approach is vital in managing their condition [5]. Usually, ECV is done for patients whose heart’s pumping ability is not steady, making them hemodynamically unstable. However, it is also quite common to use this method for patients who are stable but have recently started experiencing episodes of AFib, especially when these patients are young and do not have any underlying heart structure problems [6]. However, trying to get the heart back into its regular rhythm (known as a rhythm control strategy) using cardioversion does not consistently show the same survival benefits compared to a strategy that focuses on keeping the heart rate steady, even for younger patients or those with heart failure. Instead, cardioversion can raise the risk of blood clot-related complications, both in urgent and planned cases. Research indicates that up to 6.4% of ischemic strokes in individuals with AFib occur within 30 days after cardioversion [7]. There are two main approaches to lowering the risk of strokes after cardioversion. For patients who have had AFib for more than 48 hours, the usual method is to administer blood thinners for a minimum of three weeks before and four weeks after the cardioversion. A well-established strategy that has been used in the last decade involves using a technique called transoesophageal echocardiography (TEE) to decide when to perform the cardioversion. TEE is used to check if there are any blood clots in the left atrium before the procedure. This allows for cardioversion to be done earlier, and the duration of taking blood thinners before the procedure becomes shorter [8].

## Review

Methodology

In association with all authors, databases such as PubMed Central, MEDLINE, and PubMed were searched using various combinations of Transesophageal Echocardiography and Cardioversion. A total of 712 papers were identified, and a free full-text filter and a humans-only filter were applied to yield 212 papers. The following search strategy was selected based on the medical subject headings (MeSH) vocabulary: (“Echocardiography, Transesophageal”[Majr]) AND (“Electric Countershock”[Mesh]). A total of 177 papers were identified, and a free full-text filter and a humans-only filter were applied to yield 43 papers. No time limits were set. We selected all types of human studies that were published in English for which full text was available. Articles were excluded if the full text could not be retrieved if they did not involve humans. Duplicate publications and gray literature were also excluded. All articles underwent screening, and any disagreements among the authors were resolved through discussion until a consensus was reached. After discussion among all the authors, a total of 36 studies were unanimously included in the review.

Indications

AFib and Atrial Flutter (AF)

AFib patients are commonly electrically cardioverted to normal sinus rhythm to relieve symptoms, enhance cardiac function, and possibly lower the risk of cardioembolism [1]. To effectively treat symptomatic AFib and AF in patients who require rhythm control, cardioversion can be accomplished either through synchronized direct current (DC) electrical shock or anti-arrhythmic medications [5]. PCV is less complicated than ECV and it does not require sedation or backup anesthesia; however, it has a success rate of only about 50% as well as potential adverse effects from anti-arrhythmic medications. Although occasionally experiencing sinus asystole that may require external pacing, ECV has a better success rate of 80%-89%. If PCV fails to re-establish sinus rhythm, ECV can be used with success [9]. Indications for urgent ECV in patients with AFib include active myocardial ischemia (MI), significant hypotension, acute heart failure (HF), and the presence of accessory pathways with pre-excitation. Elective cardioversion for AFib may be performed as part of a long-term rhythm control strategy, especially for symptomatic paroxysmal AFib [10].

Ventricular Tachycardia (VTach) and Supraventricular Tachycardia (SVT)

Emergency ECV is reserved for unstable VTach causing hemodynamic compromise. If anti-arrhythmic treatment and vagotonic maneuvers such as carotid sinus massage are unsuccessful for sustained SVT, ECV should be considered as the next step [11].

Mechanism of thromboembolism post-cardioversion

**Figure 1:**
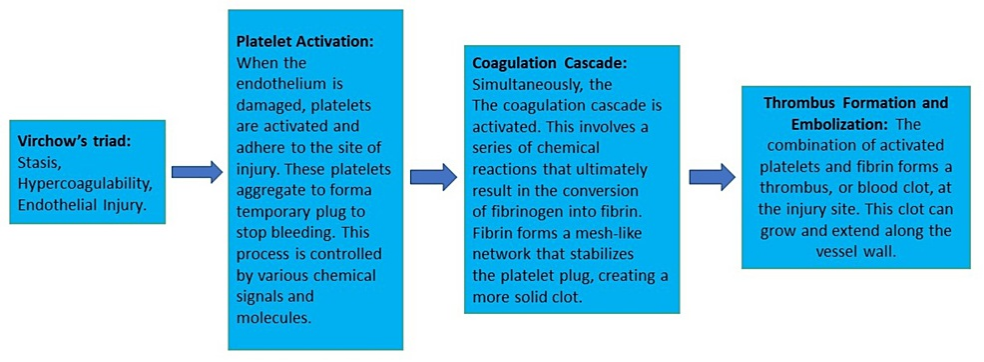
Virchow’s triad of coagulation. Image credits: Yashaswi Patel and Priyansh Patel.

Thromboembolism following cardioversion has been recognized as a significant concern, particularly in patients with congestive HF, which can lead to a high fatality rate, affecting up to 30% of patients [12]. The formation of intracardiac thrombosis, mainly within the left ventricle and atrium, is influenced by Virchow’s triad factors (stasis, damage to the endothelial lining, and increased coagulation tendency). Various factors contribute to thrombus formation, such as AFib, irregularities in the heart’s inner lining, atrial enlargement due to valvular pathologies, and conditions following MI [13]. Conditions such as low cardiac output, unusual blood flow patterns in dilated heart chambers, and weakened heart contractions can promote intracardiac thrombus formation and subsequent thromboembolism [12]. TEE is highly effective in identifying thrombi, particularly in the left atrial appendage (LAA), where clot formation is a significant risk, especially in cases of AFib. The procedure aids in detecting organized thrombi and spontaneous contrast in the left atrium, which can predict thrombus formation. Examining blood flow velocity in the LAA is critical, as velocities below 27 cm/second indicate an increased risk of thrombus [14]. Figure 1 shows Virchow’s triad and the mechanism of formation of thrombus.

**Figure 2:**
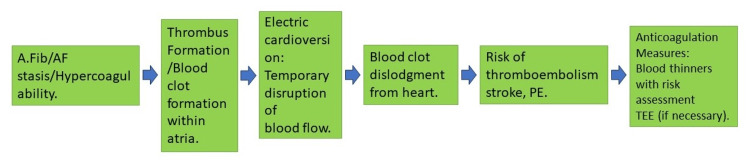
Mechanism of TEE post-cardioversion. A.Fib: atrial fibrillation; AF: atrial flutter; PE: pulmonary embolism, TEE: thromboembolic event Image credits: Yashaswi Patel and Priyansh Patel.

ECV is a well-established treatment for AFib, a common arrhythmia with an independent association with thromboembolism, leading to complications such as stroke due to clot formation in the left atrium. The ECV procedure carries a risk of dislodging clots, leading to systemic embolization. This risk has been attributed to the dislodgment of existing atrial thrombi during the resumption of atrial contractions in sinus rhythm [15]. TEE is more sensitive than transthoracic echocardiography (TTE) for detecting left atrial thrombus and spontaneous echo contrast, which indicates thromboembolic risk. Therefore, TEE can be a reliable method for screening patients before ECV. Interestingly, recent observations suggest that thromboembolism post-cardioversion may be more related to the effects of cardioversion on atrial function than the dislodgment of pre-existing thrombi [16]. The temporary impairment of atrial function increases the short-term risk of thrombus formation. Extensive registry data indicate a significantly increased risk of thromboembolic events within 30 days following cardioversion in patients not adequately anticoagulated. This has led to the establishment of prophylactic anticoagulation as a standard practice in cardioversion procedures. The sudden recovery of atrial systole during cardioversion is believed to trigger embolic events due to the migration of clots from the left atrium [17]. Figure 2 shows the mechanism of TEE following ECV.

Modalities to prevent thromboembolism

Two methods for preventing thromboembolism in patients undergoing cardioversion are anticoagulation and TEE [18,19].

Anticoagulation

Reducing the risk of thromboembolic events through anticoagulation is crucial in managing patients with AFib undergoing cardioversion. It is considered a cornerstone of peri-cardioversion management [18]. Several anticoagulation agents are available, including vitamin K antagonists (VKAs, such as warfarin), heparin, direct thrombin inhibitors (DTIs), and Factor Xa (FXa) inhibitors. Furthermore, evidence suggests that oral DTI and FXa inhibitors are effective for patients who need cardioversion. These inhibitors have advantages over VKAs and parenteral heparins [20]. The use of direct oral anticoagulation (DOAC) during cardioversion has several advantages, including faster therapeutic anticoagulation and reduced time to cardioversion. It also avoids the need for peri-procedural parenteral bridging and improves patient satisfaction when compared to VKAs. Multiple studies have reported shorter times to cardioversion with dabigatran and rivaroxaban, which can reduce delays in care and potentially lower costs [19].

TEE

Practice guidelines recommend using TEE to rule out the presence of a thrombus in the left atrium or LAA. TEE allows for immediate cardioversion in patients without a detectable thrombus, avoiding three weeks of pre-cardioversion anticoagulation [19]. The 2019 American Heart Association/American College of Cardiology/Heart Rhythm Society (AHA/ACC/HRS) guidelines for the management of patients with AFib to prevent thromboembolism in patients undergoing electrical and pharmacological cardioversion are shown in Table 1 [21].

**Table 1:** Recommendations of the American Heart Association for the prevention of thromboembolism in patients undergoing cardioversion. The necessary permissions were obtained from the original publishers [21]. AFib: atrial fibrillation; AF: atrial flutter; FXa inhibitor: Factor Xa inhibitor; TEE: transesophageal echocardiography; LAA: left atrial appendage

Recommendation of AHA for prevention of thromboembolism in patients undergoing cardioversion [21]	
Class I, level of evidence B-R	Anticoagulation with warfarin, FXa inhibitor, or direct thrombin inhibitor is recommended for at least 3 weeks prior and a minimum of 4 weeks after cardioversion for patients with AFib or AF of 48 hours duration or longer, or if the duration of AFib is unknown. This recommendation is regardless of the CHA_2_DS_2_-VASc score or method of cardioversion
Class I, level of evidence C	Immediate cardioversion is warranted for patients with hemodynamic instability due to AFib or AF of more than 48 hours duration or unknown duration. Anticoagulation should be initiated as soon as possible and continued for at least 4 weeks after cardioversion
Class I, level of evidence C-EO	Long-term anticoagulation therapy after cardioversion for AFib of any duration should be based on the thromboembolic risk profile and bleeding risk profile
Class IIa, level of Evidence B-NR	AFib or AF patients of less than 48 hours duration with CHA_2_DS_2_-VASc score of ≥2 in men and ≥3 in women can be administered heparin, FXa inhibitor, or direct thrombin inhibitor as soon as possible before cardioversion, followed by long-term anticoagulation therapy
Class IIa, level of evidence B	TEE can be performed before cardioversion if AFib or AF patients of 48 hours duration or longer or unknown have not been anticoagulated for the preceding 3 weeks. Cardioversion can be done if no left atrial thrombus is identified, including LAA, given that before TEE, anticoagulation was achieved and will be maintained for at least 4 weeks after cardioversion
Class IIb, level of evidence B-NR	AFib or AF patients of less than 48 hours duration with CHA_2_DS_2_-VASc score of 0 in men and 1 in women can be administered heparin, FXa inhibitor, or direct thrombin inhibitor versus no anticoagulation therapy, may be considered before cardioversion, also without the need for post-cardioversion anticoagulation

Role of TEE in preventing thromboembolism

TEE is a non-invasive method for detailed imaging of the heart using ultrasound. Current guidelines use TEE in patients who have AFib longer than 48 hours without anticoagulation as this is considered the time during which LAA thrombi can form and atrial stunning is likely to occur after cardioversion, increasing the risk of thromboembolism [22]. While it offers the ability to directly visualize thrombi in the LAA, the actual utility of routine use before cardioversion to prevent thromboembolism in AFib patients who have undergone adequate anticoagulation is still evolving [23]. Despite recommendations against TEE in adequately anticoagulated patients, in practice many clinicians choose to evaluate patients with TEE regardless of anticoagulation status when they have an increased risk of thromboembolism [24].

This expansion of the clinical use of TEE is often due to the experiences in clinical outcomes for ablation centers [25] and the presence of thrombi on TEE despite achieving three weeks of anticoagulation in practice [26,27]. A study from McGill University Health Center showed that a small proportion of patients had LAA thrombi on TEE with three weeks of anticoagulation, with patients on DOAC having fewer thrombus instances compared to those on warfarin [28]. Similarly, a retrospective study from 2022 found that almost half of patients still had LAA thrombi after three weeks of anticoagulation therapy [13]. This study reported a higher incidence of thromboembolic events in patients treated with warfarin, but approximately a fourth of the patients on DOAC had LAA thrombi as well [13].

Conversely, while TEE has demonstrated utility in detecting LAA thrombi, a well-known risk factor for stroke, a 2021 observational multicenter study observed no evidence of thromboembolic events in patients adequately treated with anticoagulation (apixaban, dabigatran, and rivaroxaban) without pre-procedural TEE [29]. AFib patients on oral anticoagulation demonstrated a risk of thromboembolism below 1% overall in another study [26], and anticoagulated patients with thrombi discovered on TEE experienced no embolic events after cardioversion in other studies [30,31]. Despite these findings, there is evidence of the additional utility of TEE before cardioversion in patients with certain risk factors [29].

The research on risk assessment tools for different populations is further defining the role of TEE in preventing thromboembolism in cardioversion patients. Among the risk factors identified is the discovery of the value of speckle-tracking analysis of the left atrium wall in predicting thrombi [25]. Furthermore, a multicenter prospective observational study sourced from the left atrial thrombus TEE (LATTEE) registry highlighted that younger AFib patients with a non-paroxysmal AFib, reduced left ventricular ejection fraction (LVEF), and those treated with VKAs had a higher risk of developing LAA thrombi [32]. The CLOTS-AF scoring system considers additional factors such as creatinine, LVEF, left ventricular overload, tricuspid annular plane for systolic excursion (TAPSE), stroke, AFib rhythm for high-risk individuals with unclear AFib duration who require deferment of cardioversion in favor of anticoagulation versus low-risk candidates who are appropriate for immediate TEE-guided cardioversion [33]. The role of TEE also appears to differ with race and ethnicity, as a study from China found that it was reasonable to perform TEE before treatment despite anticoagulant therapy or low CHA2DS2-VASc score due to increased risk of embolism in Asian populations [34].

In addition to clinical considerations, the diagnostic features of TEE can be further enhanced by using contrast, significantly improving the detection of LAA thrombi and early signs such as arterial smoke or sludge [22,35]. Three-dimensional TEE with color and pulsed wave Doppler allows for an even more thorough evaluation of features such as LAA emptying velocities and difficult-to-see thrombi [36]. Additionally, a 2022 study revealed that using LVEF in tandem with the CHA2DS2-VASc scoring model enhanced the prediction of LAA thrombi in non-high-risk patients [37]. Other factors correlating with an overall high thromboembolic risk that can be identified on TEE are left ventricular hypertrophy, peak antegrade flow from the LAA, and complex aortic plaque [38].

Limitations

Acknowledging the intricate and nuanced role of TEE in thromboembolism prevention after cardioversion, this review is influenced by several significant factors. The diversity in study designs, patient populations, and methodologies among the selected studies introduces potential sources of bias and variability. This heterogeneity poses challenges to drawing universally applicable conclusions and extending findings to broader clinical contexts. Additionally, a disparity exists between recommended practices and actual clinical implementations, further complicating the generalizability of the findings. Furthermore, it is worth noting that the follow-up periods in certain studies may not have adequately captured delayed thromboembolic events, possibly leading to an underestimation of the true effectiveness of TEE-guided strategies. Moreover, the evolving landscape of risk assessment tools, including techniques such as speckle-tracking analysis, clinical scoring systems, and demographic considerations, adds another layer of complexity to interpreting TEE results. It is also important to note the multifaceted nature of risk factors across diverse populations, exemplified by variations in race and ethnicity, necessitates a nuanced approach to assessing the applicability of TEE. This nuanced approach is currently lacking. Lastly, while technical advancements enhance the diagnostic capabilities of TEE, they also introduce a potential source of bias due to their limited availability and the varying levels of expertise in their application. Despite these limitations, the amalgamation of evidence presented in this review significantly contributes to our comprehension of the role of TEE in preventing thromboembolism post-cardioversion. The review underscores the urgency for more standardized research methodologies and prospective studies to further clarify its genuine clinical impact.

## Conclusions

TEE plays a vital role in thromboembolism prevention during AFib and AF cardioversion. TEE’s heightened sensitivity in detecting intracardiac thrombi, particularly within the LAA, underscores its reliability as a screening tool. While anticoagulation is paramount, TEE offers distinct advantages for immediate cardioversion cases and elevated thromboembolic risk situations. However, the evolving landscape of the role of TEE suggests that further research is warranted. Tailoring risk assessment tools to diverse populations and understanding the nuances of TEE’s contribution remain areas of exploration. Clinicians must judiciously integrate TEE, considering individual risk factors and evolving guidelines, into the pre-cardioversion protocol. The potential augmentation of TEE’s diagnostic capabilities using contrast agents also warrants investigation. As we advance, TEE’s role in optimizing thromboembolism prevention in AFib and AF patients undergoing cardioversion will continue to unfold. Further research will refine its application and strengthen its contribution to patient outcomes, reflecting the dynamic nature of cardiovascular care.
